# Addressing the spread of health-related misinformation on social networks: an opinion article

**DOI:** 10.3389/fmed.2023.1167033

**Published:** 2023-05-02

**Authors:** Maria Polyzou, David Kiefer, Xenofon Baraliakos, Philipp Sewerin

**Affiliations:** ^1^Ruhr-University Bochum, Rheumazentrum Ruhrgebiet, Herne, Germany; ^2^Department of Rheumatology & Hiller Research Unit, University Hospital Düsseldorf, Düsseldorf, Germany

**Keywords:** misinformation, social networks, health sector, rheumatology, COVID-19

## Abstract

This article deals with the spread of misinformation in a general context and specifically in the health sector. It presents a theoretical view of the problem and analyzes its characteristics with a focus on medicine and mainly rheumatology. Finally, conclusions from the previous analysis are formulated as well as suggestions for reducing the dimensions of the problem in the health sector.

## Introduction

The improvement of technology used on social media and its increasing use in everyday life results in a rapid increase of online social networks and users. Over the last two decades, the users of social networks, from all social groups regardless of gender or age, have increased, to seek and share health-related information. Moreover, social networks became very helpful to health professionals and organizations that use them to advise on healthy habits, medical information, and disease prevention. On the opposite, social networks could potentially try to spread misinformation inadvertently or with different financial and social motivations; a problem that has attracted lots of attention recently (1, 2).

For the spread of “false” information, researchers and increasingly even the general population use the term “fake news,” which is not sufficient to cover the complexity of the phenomenon. There are concepts proposed by researchers in an attempt to explain and analyze phenomena such as misinformation, disinformation, counterfeit news, propaganda, information disorder, post-truth, etc. According to the Reuters Institute Digital News Report difficulties in defining “fake news” are based on the fact that three different types of news are involved, which are mentioned below. This report identifies for the first time the quality characteristics that distinguish online news, as perceived by users, such as accuracy and reliability, helping to understand complex issues, communicating strong opinions, and providing entertaining content (3).

## General analysis on the spread of misinformation

Understanding the phenomenon of misinformation is a basic condition for dealing with it. For this reason, a brief description of the basic characteristics (typology, motivations, sources, creation, transmission, reproduction, etc.) behind the production and dissemination of misinformation or “*fake news”* will be provided below.

In a general conceptual framework and using the dimensions of harm and falseness, the differences between the three types of information are (4):

• **Mis-information**, when false information is shared without the intention of causing harm.

• **Dis-information**, when false information is intentionally shared with the intent to cause harm.

• **Mal-information**, when genuine information is shared to cause harm, often by bringing information designed to remain private into the public sphere.

The main motivations behind the creation of “fake news” can be **economic**, **ideological**, or **political**. There is also a special category of “fake news” that is created for **humorous reasons**, to entertain, or simply provoke. In some cases, the creation of “fake news” serves **social reasons**, such as a person’s desire to belong to a certain group online, as well as **psychological reasons**, related to the person’s desire to gain prestige and authority (4). Usually, political parties, secret services, news organizations, also individuals, who adhere to a certain ideology or support certain politicians, have political or ideological motives.

The sources behind fake news for financial gain vary from Public Relations (PR) companies, and advertising agencies to “manufactured” news media. Especially the latter is based on sharing content to make immediate profit from online advertising, while they are mostly not interested in “building” a name and gaining the trust of the people (4, 5).

The above separation is considered necessary as a message spread on the internet can be reproduced countless times, by different actors and thus also with different motivations. For the correct treatment and reaction to the messages spread, their understanding as well as the way they are consumed and interpreted is equally important. The production of a message is divided initially into its creation followed by its transformation into a so-called news product. Accordingly, the public is not a passive recipient of information, as everyone interprets the information they will receive according to their social, and economic level, political beliefs, and personal experiences, while they can also become transmitters, announcing the news (6).

“Fake news” can be co-produced by the public, as the “falseness” of news depends primarily on whether the public perceives the lie as truth. Until the public is convinced that a fake news story is true, the process of deception is not complete. However, fake news may continue to be shared, either because the public wants to express their agreement or disagreement with it or to inform that it is invented “fake news” (7). The motivation of users who republish fake news is mostly not financial, since they most often do not gain anything from reproducing a news story. In contrast, users are usually socially and psychologically motivated and seek to express their opinions with their shares, connect with like-minded people and gain the sympathy, respect, and recognition of others.

Understanding the phenomenon of the spread and acceptance of “fake news” as true is easier by examining the so-called “**echo chambers**.” Users tend to select and share content related to a particular narrative and ignore the rest. Most often the information is obtained from close social sources of the user, which helps to “legitimize” the fake information spreading on social media or from people who belong to the same echo chamber. The way the news feed appears on users’ social media homepages results in selective exposure to certain types of news (8, 9).

Therefore, social media users tend to form ideologically homogeneous online communities (echo chambers), in which narratives that satisfy and confirm their perceptions and beliefs are repeated, creating the “echo” effect. The effect of “echo” combined with psychological factors such as “social credibility” and “frequency heuristics” facilitate the process by which people consume and believe fake news “**social credibility**” is defined as the perception that something is reliable when others have judged it as such and the “**frequency heuristic**” the ability of consumers to favor information they hear often, even if it is false. Each person has fixed opinions on specific issues which influence whether they will believe specific news or not.

In social networks, the credibility and increased exposure of “fake news” are persistent over a long period. This fact, combined with the frequent appearance in many different sources and the ease with which the human brain processes the message, increases the chances that users will perceive it as true (10).

## Spread of health-related misinformation

In general, extensive dissemination of misleading or false information via social networks or other ways is unfortunately widely spread in many sectors of daily life. For instance, seen in environmental issues or campaigns about migrants and minority groups. The same happens also in the field of medicine, where false information leads potentially to a serious threat to health and safety, especially during the time of a pandemic.

Effective and valid health communication is widely recognized as a key element in addressing health issues. An important aspect of health communication is concerning wider public health, as it can lead the public to different conclusions on the very critical issue of public health and its protection and development as a social good.

Nowadays, it is widely accepted that one of the most important and determining factors of health are social factors and economic status, and less individual behavior. In this context, greater effort is being made to focus broader public health campaigns on influencing individual behavior, as this is considered a more cost-effective approach to improving health. However, when a public health policy is ineffective or harmful, entire populations can be at serious health risk. Hence, the fundamental problem that should concern physicians and scientists equally, is related to the possibility of maximizing the benefits resulting from the spread of information related to the health sector, protecting society from misinformation and disinformation and the consequent negative effects on public health.

After the appearance of each new disease, especially when it has the characteristics of a pandemic, misinformation is spread on social media about the reliability of new drugs and vaccines proposed to treat or prevent it. The peak of misinformation was seen in the last 2 years and was related to the treatment of COVID-19. Misinformation is mainly due to the uncertainty surrounding the etiology and the consequences of each new disease, while it is reinforced by misaligned institutional communication. The rapid spread of misinformation on social networks disturbs the authenticity balance of the communication systems and pushes governments to curb the spread of misinformation to avoid the risk of behaviors that are potentially harmful to the population (7).

Recently, in the era of the pandemic of COVID-19, the prolific rise of medical misinformation was a pressing public health concern, since caused confusion, sowed mistrust, harmed people’s health, and undermined public health efforts. Misinformation in many cases worldwide delayed people’s access to care which negatively affected their health status. Such information worsened the existing fear about the effectiveness and consequences of vaccines and limit their uptake by a population. These developments can be characterized as negative for the evolution of the pandemic, since they reduced the population level of indirect protection from the vaccine, or even they prevented herd immunity that could eliminate the virus earlier (11).

It is worth to be noted that many public health professionals were surprised since they were completely unprepared to face the misinformation surrounding COVID-19 and the subsequent vaccine rollout. Unprecedented conditions understandably created increased needs for information. Nowadays, medicine has a truth problem, since it has been identified that medical misinformation constitutes a major public health threat, and many professional societies, including the American Medical Association, have called for action to fight it (11, 12).

During health crises, the overproduction of data and information from multiple sources, the quality of the information, and the spread of information create social and health-related impacts. This phenomenon, called an “*infodemic*,” involves false or misleading information mainly in social media during a disease outbreak. This impact is mostly negative because it causes confusion and risk-taking behaviors that can harm individual healthor even entire populations. In addition, it leads to mistrust in health authorities and undermines the public health response (13).

Medical misinformation is a current social problem that also **affects rheumatologists**, whereby the COVID-19 pandemic has amplified the dimensions of the problem (14). In the scientific field of rheumatology, research results have shown that misinformation has corresponding effects on the treatment progress of many patients. Patients stopped taking medicines either due to fear and lack of adequate information or due to misinformation (15).

Although rheumatologists are confronted daily with misinformation about both general health issues and rheumatic and musculoskeletal conditions, publications on misinformation in rheumatology are limited (16). In a study, the impact of a national current affairs television program about the association between osteonecrosis of the jaw and bisphosphonates on subsequent prescription use, fractures, and deaths has been examined (17). The study concluded that, although patients need to be informed about the risks of medication, the information provided by the media was unbalanced and could potentially do more harm than good.

In a study that concerned Spain, the content of a random sample of original tweets on Twitter, mainly about rheumatoid arthritis and osteoarthritis, 1,093 were classified as medical and 421 as non-medical has been analyzed (18). A small rate of false information was found (4.4%), a little higher for therapeutics (5.8%). False information received fewer retweets and likes than accurate information, which suggests regulation by the Twitter community.

Given that some categories of patients, such as rheumatic patients, face a set of challenges related to whether they are at greater risk for complications coming from COVID-19 or other illnesses, these patients are more susceptible to misinformation. In a study conducted in Netherlands during the two peak months of the COVID-19 outbreak, results showed that people with the inflammatory rheumatic disease were more worried about getting infected and more stressed than people without an inflammatory rheumatic disease (19). Another study concluded that in rheumatic patients in which immunosuppressive medications had been used, the uncertainty had been increased about the risk of COVID-19 infection, as well as whether arthritis medications should be continued during the COVID-19 outbreak (20).

As a consequence, after the COVID-19 pandemic, patients trust the scientists in the sector of health less or could obtain their information from less reliable sources, often inaccurate sources on social media (15). This has created a challenge in the delivery of good medical care.

As a result of a mixed methods study published in July 2021 in ACR Open Rheumatology, Birru Talabi et al. reported that 80% of pregnant patients refused to continue even safe medication in preparation for pregnancy or during pregnancy, although they did not fully recover from their symptoms. Furthermore, they reported that pregnant patients developed an undefined trust in their doctors due to conflicting advice about treatment during pregnancy (15).

Finally, some recent articles are demonstrated that social media can play an important role in disseminating clinical expertise, facilitating communication among researchers, and engaging with patients. These articles highlight some of the pitfalls involved in the widespread adoption of social media usage in medicine and the most effective ways in which to combat these (21).

## Conclusion—Proposals

The volume of available health information is vastly growing and the volume of published scientific health information has accelerated at an unprecedented rate in the past 2 years not only but mostly due to the pandemic. The number of relevant publications and scientific papers is growing at a rate of more than 8% per year and more than 1 million new publications are registered in PubMed annually (22).

The increase in misinformation creates significant problems, which mainly concern the acceptance of medical advice by patients and the population in general, as well as hindering the practice of the medical profession (23). As already mentioned in many surveys that have been carried out, a large proportion of the population appears hesitant toward vaccines, while the refusal to accept vaccines increases with misinformation spread for example on social media. Additionally, before the COVID-19 pandemic, 1 in 4 physicians reported being personally attacked on social media, while during the COVID-19 pandemic, nearly 60% of scientists and physicians surveyed had experienced attacks on their credibility and 15% had received threats to their lives (22).

It is quite understandable that misinformation is harmful to individual and public health and therefore it seems necessary, that a healthier information environment is created. Despite the large increase in misinformation in the health industry, studies addressing the phenomenon at least in rheumatology are not increasing accordingly. The small number of available studies does not favor the effective management of this important topic in rheumatology.

In general, significant efforts to address health misinformation needs to be undertaken by responsible health agencies and competent government agencies with the assistance of the citizens. This includes the following actions:

• Health professionals and trusted community members, such as faith leaders, professors, and politicians, have to speak directly to their communities to address related questions whereby necessarily scientific and above all validated sources have to be applied (e.g., about pandemics, the usefulness of vaccines, etc.). Moreover, the use of technology and media platforms by health professionals to share accurate health information with the public may be very important.
• Understanding each patient’s knowledge, beliefs, and values is very important so that health professionals can overcome any resistance of patients to accept the appropriate treatment, again supported by validated and scientifically based information.
• The relevant Ministry of Health and other authorities involved should inform the public about public health by highlighting the dangers of misinformation using persuasive arguments in collaboration with trusted messengers.
• More resources (staff and funding) need to be devoted directly to media organizations to spot relevant misinformation on social media.
• Addressing health misinformation will require a whole-of-society effort. Everyone should seek to be informed by official organizations and services about dealing with diseases and especially pandemics.

In addition, health professionals need to better understand the factors that lead people to accept misinformation. Although there are not in international literature descriptions of specific interventions at the level of the patient-physician relationship, identifying these factors may help physicians when counseling patients about misinformation, while more research is needed on misinformation diffusion in the field of rheumatology.

In the middle of a global health crisis, it is distressing to read the following statement of the Federation of State Medical Boards (FSMB) (Washington, D.C., USA, July 29, 2021) in response to a dramatic increase in the dissemination of COVID-19 vaccine misinformation by physicians and other health professionals on social media: “*Physicians who generate and spread COVID-19 vaccine misinformation or disinformation are risking disciplinary action by state medical boards, including the suspension or revocation of their medical license*” (24).

We think that both in the current period of the health crisis due to COVID-19 and in general, scientists and physicians have a special power and capability to persuade society, due to their specialized knowledge and training, but also a high degree of public trust. Along with that, they have also a big responsibility to practice medicine and society in general which should be used to avoid the distribution or even sharing of misinformation.

## Author contributions

MP, DK, XB, and PS wrote the manuscript. All authors contributed in the finalization of the draft and approved the final version.
